# Unraveling the enigma of eosinophilic gastroenteritis: a rare case of multisystem involvement in an elderly patient

**DOI:** 10.1093/omcr/omaf302

**Published:** 2026-02-18

**Authors:** Henna A Qadri, Amit Sagar, Asad Zaman, Abdul Rafae Faisal, Pramod Singh, Garcia Chiroles Patiricia

**Affiliations:** Department of Internal Medicine, Memorial Hospital, 7800 Sheridan St, Pembroke Pines, FL 33024, United States of America; Department of Medicine,FAU Charles E. Schmidt College of Medicine, 777 Glades Road BC-71, Boca Raton, FL 33431, United States of America; Department of Internal Medicine, CMH Institute of Medical Sciences, 5CWP+V7, Naseem Hayath Road, Cantt Area, Punjab, Pia Colony, Multan, Pakistan; Department of Internal Medicine, CMH Institute of Medical Sciences, 5CWP+V7, Naseem Hayath Road, Cantt Area, Punjab, Pia Colony, Multan, Pakistan; Department of Internal Medicine, Barhabise Primary Health Care Centre, Bahrabise Municipality-09, Ramche, Sindhupalchowk, Bagmati, Kathamandu, 45300 Nepal; Department of Internal Medicine, Memorial Hospital, 7800 Sheridan St, Pembroke Pines, FL 33024, United States of America

**Keywords:** eosinophilic gastroenteritis, eosinophilic infiltration, gastrointestinal disorders, peripheral eosinophilia, multisystem involvement

## Abstract

Eosinophilic gastroenteritis (EG), the most common form of eosinophilic gastrointestinal disease, primarily affects the stomach and small intestine. This retrospective case report, following CARE guidelines, analyzed clinical data and imaging. A 77-year-old male presented with substernal chest pain, weakness, appetite loss, and weight loss. CBC showed leukocytosis with marked eosinophilia. Abdominal CT revealed thickened jejunal loops. Biopsies confirmed eosinophilic infiltration in the duodenum and ileum, diagnosing EG. The patient was treated with prednisone, leading to significant improvement. This case highlights the importance of recognizing EG in patients with unexplained GI symptoms and eosinophilia for timely diagnosis and effective management.

## Introduction

Eosinophilic gastrointestinal disease (EGIDs) encompasses a group of rare disorders characterized by the atypical infiltration of eosinophils into any section of the gastrointestinal (GI) tract, excluding known secondary causes such as drugs, parasitic infestations, and neoplasms. These disorders include eosinophilic esophagitis, eosinophilic gastritis, eosinophilic gastroenteritis, eosinophilic enteritis (EE), and eosinophilic colitis [1]. The most common of these is eosinophilic gastroenteritis (EGE) [2]. Although no accurate epidemiologic data are available, the incidence of EG is estimated to be approximately 1–30/100000 [3]. Based on the histopathological findings of eosinophilic infiltration, it can be divided into three types: mucosal (40%–70%), muscularis (10%–30%), and sub serosal (10%–15%) [4]. Clinical manifestations vary greatly, depending on which layer and part of the GI tract are predominantly affected. The stomach and small intestine are the most commonly affected areas [5]. It is diagnosed in the biopsies taken during endoscopic examination to the patients with abdominal pain and chronic diarrhea [6].

## Case presentation

**Figure 1:**
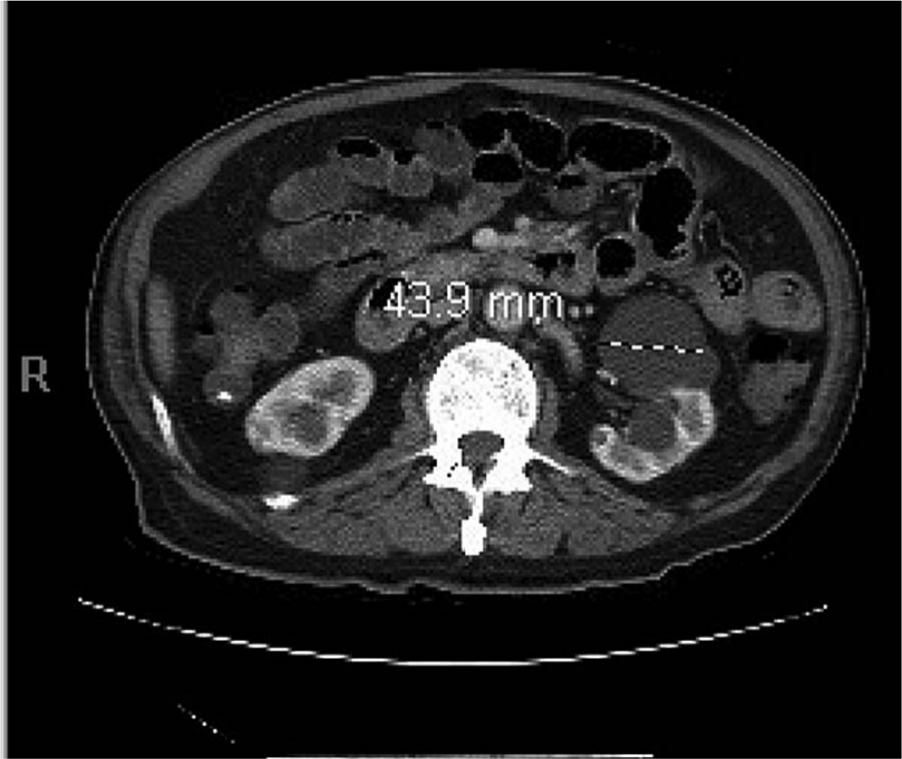
Abdominal CT scan showing thickened jejunal loops in the right upper quadrant, suggestive of small bowel involvement in eosinophilic gastroenteritis.

**Figure 2:**
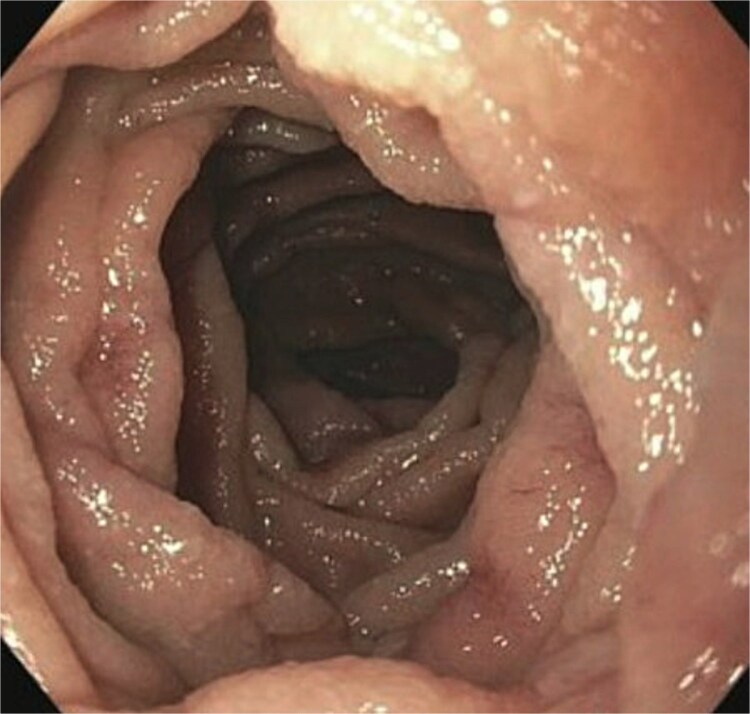
Histological section of the duodenum revealing dense eosinophilic infiltration in the mucosa (H&E stain, high power view), consistent with eosinophilic gastroenteritis.

**Figure 3:**
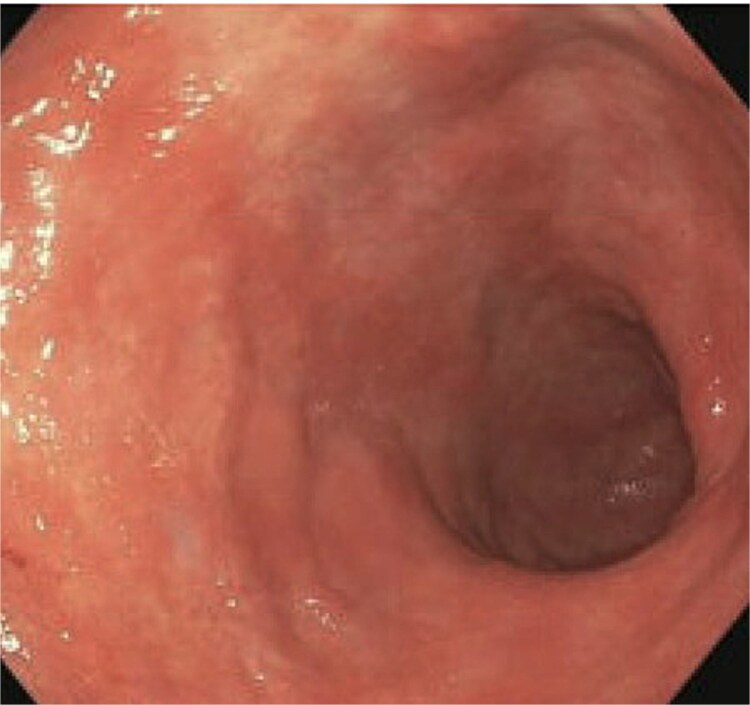
Ileal biopsy showing marked eosinophilic infiltration and disrupted mucosal architecture (H&E stain), supporting the diagnosis of eosinophilic gastroenteritis.

**Figure 4:**
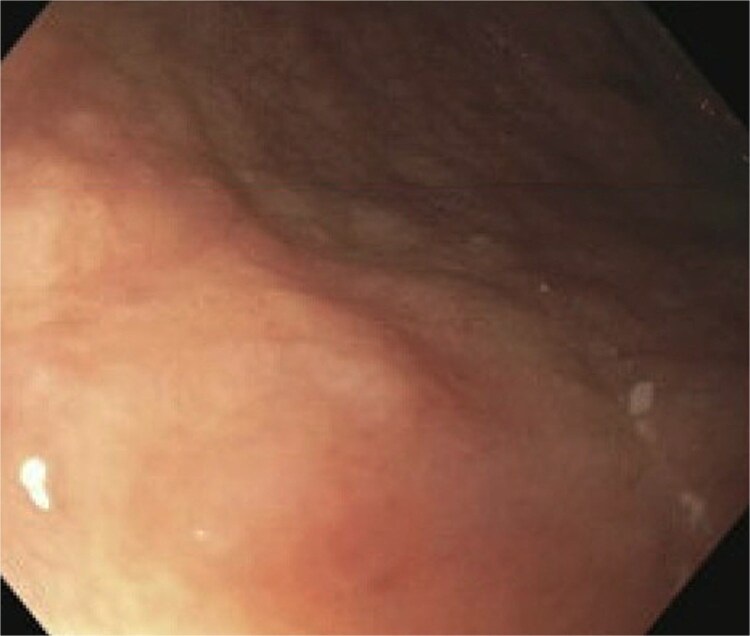
Gastric mucosa biopsy showing no evidence of intestinal metaplasia.

**Figure 5:**
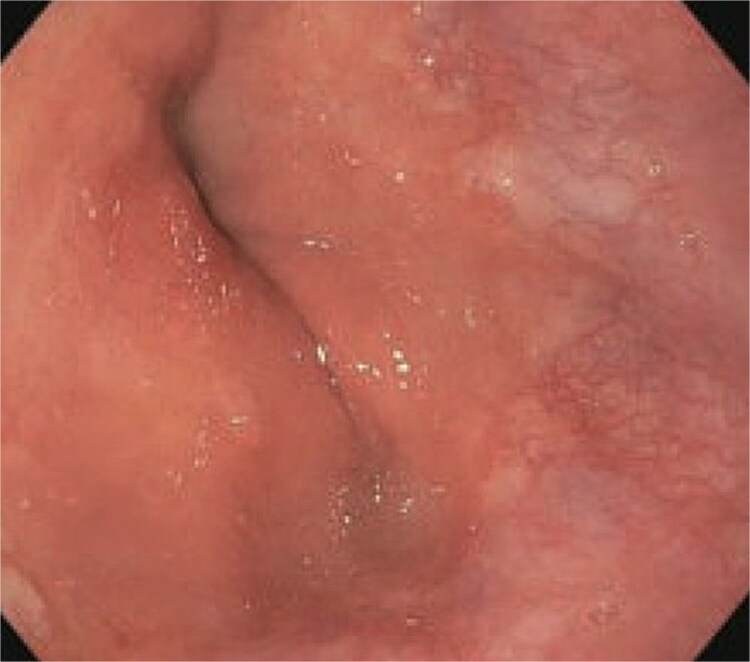
Terminal ileum biopsy with diffuse and prominent eosinophilic infiltration within the lamina propria and intraepithelial layers, consistent with eosinophilic gastroenteritis.

**Figure 6:**
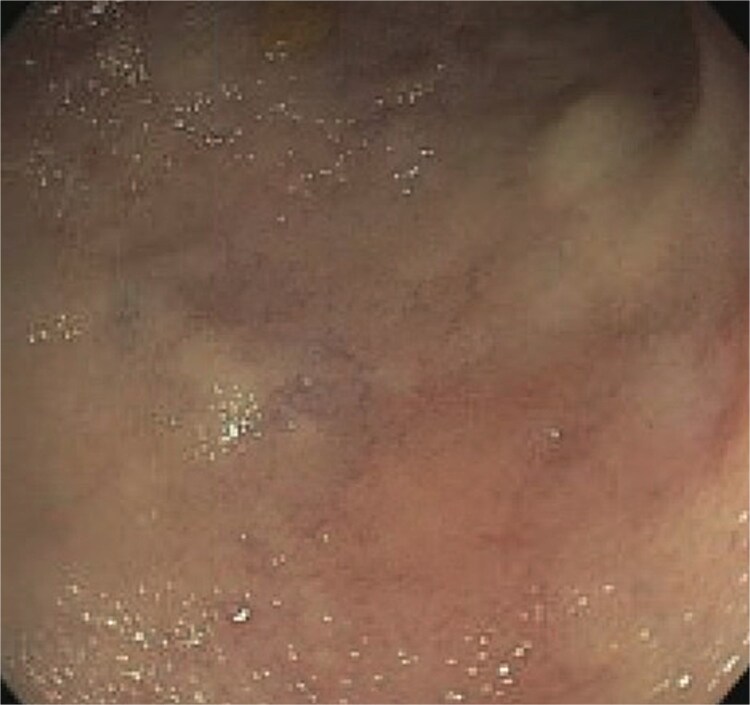
Descending colon biopsy demonstrating marked eosinophilic infiltration in the lamina propria and intraepithelial compartments, supporting a diagnosis of eosinophilic involvement of the colon.

**Figure 7:**
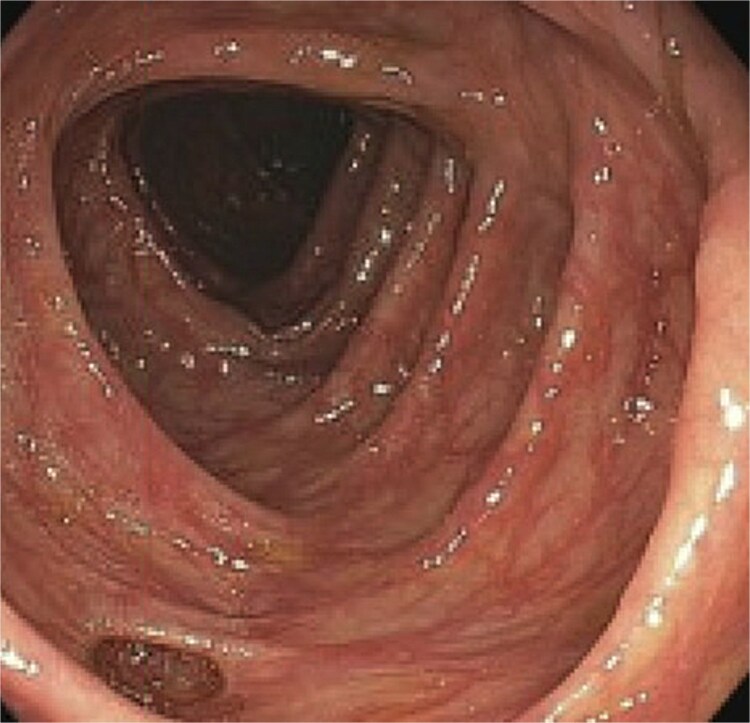
Descending colon biopsy demonstrating marked eosinophilic infiltrate in the lamina propria and intraepithelial compartments, supporting a diagnosis of of eosinophilic involvement of the colon.

**Figure 8:**
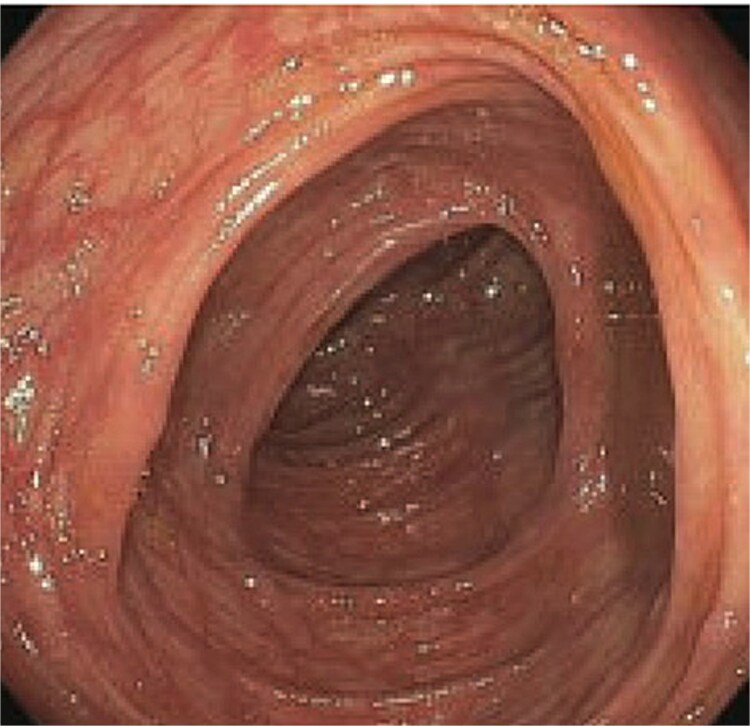
Descending colon biopsy demonstrating marked eosinophilic involvement in the lamina propria and intraepithelial compartments supporting a diagnosis of eosinophilic involvement of the colon.

A 77-year-old male presented to the emergency room with the chief complaint of substernal chest pain and generalized weakness. He had a past medical history of stroke, type II diabetes mellitus, hypercholesterolemia, and hypertension. For the past few days, the patient had intermittent episodes of sharp, non-radiating chest pain that self-resolved. He did not notice any triggers for his pain and did not have any shortness of breath or worsening of symptoms during exertion. For the past few weeks, he had a dry cough along with dysphonia and a loss of appetite for solid foods without dysphagia. He had lost about thirty pounds in the three months before the presentation. Moreover, he denied any association between his chest pain and food consumption. He reports an allergy to ACE inhibitors, with use resulting in angioedema. Physical examinations revealed no significant findings. Troponin levels were normal. CBC demonstrated elevated WBC count (14.2/μl), red cell distribution (14.6 million/mcL), and blood urea nitrogen (BUN) (28 mg/dl) (Table 1). Chest x-ray scan showed no acute findings. An EKG was performed which confirmed an elevated heart rate of 120 beats per minute, resulting from sinus tachycardia. The patient was given IV normal saline, which resolved his tachycardia, and was admitted for further evaluation. After admission, the patient stated that his chest pain had resolved. He complained of new suprapubic abdominal pain on the 1st day of admission and was treated with Rocephin empirically, but urinalysis returned normal. The patient also reported diarrhea. CBC showed worsening leukocytosis with a WBC count of 17.5/μl and marked eosinophilia of 9.22% on 2nd day of admission. BUN improved to 22 mg/dl. Echocardiogram with doppler revealed mildly decreased left ventricular systolic function with an ejection fraction of 42%, focal sclerosis of the aortic valve, and a benign 0.67 by 0.33-centimeter mass on the valve. The mass raised concern for endocarditis, but transesophageal echogram and blood cultures returned negative. Abdominal CT revealed thickening of jejunal loops in the right upper quadrant, suggestive of infectious enteritis and intramural hemorrhage. X-ray esophageal with barium showed no remarkable findings. X-ray of abdomen showed colonic diverticulosis. Multiple gas distended small bowel loops were also present. CT enterography revealed multiple wall thickenings of jejunal loops and proximal sigmoid colon along with diverticulosis (Fig. 1). Esophagogastroduodenoscopy (EGD) revealed diffuse inflammation with hemorrhage along with erosions and congestion (edema) in duodenal bulb and through all the portions of duodenum. Infectious disease recommended ceftriaxone 2 gr IV q24 hrs. For suspected Whipple’s disease. CT arteriogram showed no pneumatosis or portal venous air ruling out chronic mesenteric ischemia. However, it did show wall thickening of small bowel loops suggesting enteritis. Infectious enteritis workup for tropheryma whipplei, bartonella, Coccidioides, and stool ovum were negative, ruling out Whipple disease. A follow-up endoscopy showed gastritis and duodenitis with hemorrhage. A colonoscopy showed terminal ileitis and segmental colitis. Biopsies from both the endoscopy and colonoscopy were done. The endoscopic biopsies demonstrate portions of duodenal and gastric mucosa, remarkable for an increased number of eosinophils in the lamina propria and the epithelium (Figs 2 and 3). The duodenum shows no evidence of villous atrophy and there is no intraepithelial lymphocytosis (immunostaining for CD3 was performed), thus excluding celiac disease (Fig. 4), while the gastric mucosa shows no evidence of intestinal metaplasia (Fig. 5). Histochemical stains for both PAS and AFB in both biopsies came out to be negative. Colonoscopy biopsy showed both terminal ileum and descending colon to have diffuse prominent infiltration of eosinophils within the lamina propria and intraepithelial without architectural distortion, granuloma formation, or chronic crypt injury, thereby excluding features consistent with inflammatory bowel disease. (Figs 6–8). The descending colon shows the additional finding of active inflammation with prominent intraepithelial neutrophils and foci of crypt abscesses. PAS and GMS stains performed on the terminal ileum and descending colon showed no evidence of fungal organisms as well as CMV immunostaining performed on the ascending colon showed no evidence of viral inclusion. The findings of both biopsies are nonspecific and are present in but not limited to allergic reactions, infection, drug reactions, inflammatory bowel disease, and eosinophilic gastroenteritis. A review of medications excluded drug-induced eosinophilia. The laboratory tests for (Anti-nuclear antibodies) ANA and anti-neutrophil cytoplasmic antibodies (ANCA) were both negative. Autoimmune and connective tissue disorders were considered; however, serologic markers including ANA and ANCA were negative, and there were no clinical features suggestive of systemic vasculitis or connective tissue disease. Thus, eosinophilic gastroenteritis was suspected, as both biopsies showed marked eosinophilia. Taken together, the diffuse and dense eosinophilic infiltration within the mucosal and submucosal layers, coupled with negative infectious and neoplastic stains, was strongly indicative of primary eosinophilic gastroenteritis rather than a secondary reactive process. This histopathologic profile, along with the patient’s peripheral eosinophilia and gastrointestinal symptoms, confirmed the diagnosis. Hence, the patient was started on oral prednisone. This was followed by a significant improvement in the patient’s symptoms. WBCs lowered to 9.3/μl and eosinophils to 0.05%. During the patient’s hospital course, his family also noticed a cognitive decline. Brain MRI scan revealed punctate acute/subacute infarct in the right cerebellum and moderate chronic microvascular ischemia with scattered punctate chronic infarcts. CT arteriogram scan of the brain and neck revealed a 5 mm partially calcified aneurysm at the junction of the terminus segment of the right internal carotid artery with the right middle cerebral artery. Patient was treated with aspirin 81 mg daily and Brillenta 90 mg q12h for this. Ultrasound venous doppler of lower bilateral showed no signs of Deep venous thrombosis. CT scan of the chest showed no acute process. Echocardiogram revealed a positive patent foramen ovale (PFO). It was suspected that the aortic valve ‘mass’ seen on the first echocardiogram may have been the embolic culprit. Thus, there were no definite valve masses and the left ventricular systolic function remained unchanged, as compared to a prior transthoracic echo study.

**Table 1:** Hematologic and biochemical laboratory parameters on admission.

Complete blood count on admission		
Complete blood count	Patient’s result	Reference ranges
White blood cells	14.2.	4.0–10.0 × 10^3^/10^9^ l
Red blood cells	14.6	4.5–5.5× 10^6^/10^9^ l
Haemoglobin	16.1	13.0–17.0 gm/dl
Haematocrit	45.6	40%–50%
Platelets	300	150–410× 10^3^/10^9^ l
Clinical Chemistry on Admission		
Blood Urea Nitrogen	28	6–24 mg/dl
Creatinine	1.15	0.74–1.35 mg/dl
Sodium	137	133–146 mmol/l
Potassium	4.5	3.5–5.3 mmol/l
Chloride	104	95–108 mmol/l
Bicarbonate	27	22–29 mmol/l
CRP	2.6	0–5 mg/dl

This table summarizes the patient’s complete blood count and clinical chemistry results obtained at hospital admission.

## Discussion

Eosinophilic gastroenteritis is a rare and heterogeneous condition characterized by eosinophilic infiltration of the GI tract. The prevalence is estimated to be between 8.4 and 28 per 100 000 individuals, with most people diagnosed between ages thirty and fifty [1, 2]. It is often associated with a history of atopic disorders, including asthma, eczema, and allergic rhinitis.

It is important to note that in elderly patients, such as in our case, the presentation of EGE may reflect a multifactorial interplay of age-related immune dysregulation, chronic comorbid conditions, and systemic inflammatory processes. Recognizing this complex background is essential for accurate diagnosis and individualized management, as EGE may be masked by symptoms arising from coexisting diseases.

The pathogenesis of EGE is not fully understood but likely involves a combination of immune dysregulation and genetic predisposition resulting in eosinophilic infiltration with elevated Th2-mediated immunity and potential allergy-related triggers. Elevated levels of IL-5 are often implicated in eosinophilia and are a potential target for therapies.

The clinical presentation of EGE is often non-specific. The most common symptoms include abdominal pain, nausea, vomiting, poor appetite, weight loss, and diarrhea. EGE can be classified by location and depth of eosinophilic infiltration, with varying symptoms for each subtype. Mucosal subtype EGE is the most common type and often leads to the aforementioned non-specific symptoms. Some patients also present with blood loss and malabsorption. Muscular subtype EGE is the second-most common type and causes bowel wall thickening, leading to gastrointestinal obstruction. This is most often described at the gastric outlet but has been reported in the ileum, jejunum, and left colon as well [2]. Serosal subtype EGE is the least common type and often leads to eosinophilic abdominal ascites along with symptoms typical of mucosal or muscular EGE.

Patients with GI symptoms resembling EGE should be worked up for secondary causes, including other autoimmune disorders (e.g. Crohn’s disease and ulcerative colitis), infection, and malignancy. Upon exclusion of secondary causes, diagnosis of EGE requires evidence of eosinophilic infiltration of the GI tract through biopsy. CBC will typically reveal elevated absolute eosinophil counts and elevated WBCs. EGD may demonstrate similarities to gastroenterocolitis and findings of mucosal erythema and friability have been reported. A CT scan may demonstrate thickening of the stomach and intestinal lining and irregular folds. Histopathologic confirmation remains the cornerstone of diagnosis. In our case, the combination of extensive mucosal eosinophilic infiltration and exclusion of secondary causes provided definitive evidence for eosinophilic gastroenteritis.

The management of EGE is focused on symptom control with reduction of inflammation. Steroids are the first-line treatment as they have been shown to inhibit eosinophilic growth factors. Prednisone 40 mg daily is typically prescribed for two weeks, followed by a taper. Antihistamines, mast cell stabilizers (sodium cromoglycate), leukotriene antagonists (montelukast), immunomodulators (azathioprine), tumor necrosis factors inhibitors (infliximab), mepolizumab, and IgE monoclonal antibody (omalizumab) have been used as well. Dietary elimination has also been shown to improve symptoms as certain individuals generate IgE antibodies to specific foods. However, the determination of these foods is difficult as many individuals demonstrate IgE antibodies on food allergy testing but have no reaction with ingestion. Furthermore, symptoms seem to return upon reintroduction of food. There are no specific guidelines for treatment of EGE however, the use of systemic corticosteroids as the first-line treatment has been recomended. The patient’s rapid clinical and hematologic response to oral prednisone (40 mg/day) reinforces the efficacy of guideline-based corticosteroid therapy. Escalation to immunomodulators or biologics was deemed unnecessary, as complete remission was achieved with corticosteroids alone.

This case provides several novel insights that expand upon previously reported cases of eosinophilic gastroenteritis. First, it illustrates that EGE can present atypically in elderly patients with multiple chronic comorbidities, an age group in which this disease is often overlooked. Second, unlike most published reports that describe EGE as a localized gastrointestinal disorder primarily affecting younger populations, our report highlights a rare presentation in an elderly patient with extensive gastrointestinal tract involvement and associated extraintestinal features. Finally, the patient’s marked clinical and hematologic improvement with corticosteroid therapy emphasizes that standard first-line treatment remains highly effective, even in complex multisystem contexts. Together, these findings extend the known demographic and clinical spectrum of EGE and reinforce the importance of maintaining diagnostic suspicion in atypical or elderly presentations.

The prognosis of EGE is variable as its severity ranges from mild and intermittent symptoms to chronic disease with complications such as strictures, malnutrition, or intestinal obstruction. Most patients respond well to dietary or pharmacological interventions, though relapses are common. There is also no established prevention strategy for EGE due to its unclear etiology. However, there are several immune pathways under investigation for treatment options. Avoidance of known allergens in sensitized individuals and management of comorbid atopic conditions may also reduce flare-ups.
